# Social Media–Driven Routes to Positive Mental Health Among Youth: Qualitative Enquiry and Concept Mapping Study

**DOI:** 10.2196/32758

**Published:** 2022-03-04

**Authors:** Janhavi Ajit Vaingankar, Rob M van Dam, Ellaisha Samari, Sherilyn Chang, Esmond Seow, Yi Chian Chua, Nan Luo, Swapna Verma, Mythily Subramaniam

**Affiliations:** 1 Research Division Institute of Mental Health Singapore Singapore; 2 Saw Swee Hock School of Public Health National University of Singapore Singapore Singapore; 3 Department of Psychosis Institute of Mental Health Singapore Singapore; 4 Institute of Mental Health Singapore Singapore; 5 Duke-National University of Singapore Graduate Medical School Singapore Singapore

**Keywords:** teens, young adults, concept mapping, mental well-being, digital media, social media, mental health, social support, mental health promotion, self-expression

## Abstract

**Background:**

Social media influence almost every aspect of our lives by facilitating instant many-to-many communication and self-expression. Recent research suggests strong negative and positive impacts of social media exposure on youth mental health; however, there has been more emphasis on harmful relationships.

**Objective:**

Given the limited research on the benefits of social media for mental health, this qualitative study explored the lived experiences of youth to understand how social media use can contribute to positive mental health among youth.

**Methods:**

Using an interpretivist epistemological approach, 25 semistructured interviews and 11 focus group discussions were conducted with male and female youth of different ethnicities (aged 15 to 24 years) residing in Singapore, who were recruited through purposive sampling from the community. We conducted inductive thematic analysis and concept mapping to address the research aims.

**Results:**

We found that youth engaged in a wide range of activities on social media from connecting with family and friends to participating in global movements, and these served as avenues for building positive mental health. Based on participants’ narratives, our analysis suggested that positive mental health among youth could be influenced by 3 features of social media consumption (connection with friends and their global community, engagement with social media content, and the value of social media as an outlet for expression). Through these, pathways leading to the following 5 positive mental health components were identified: (1) positive relationships and social capital, (2) self-concept, (3) coping, (4) happiness, and (5) other relevant aspects of mental health (for example, positivity and personal growth).

**Conclusions:**

The study results highlight the integral role of social media in the lives of today’s youth and indicate that they can offer opportunities for positive influence, personal expression, and social support, thus contributing to positive mental health among youth. The findings of our research can be applied to optimize engagement with youth through social media and enhance the digital modes of mental health promotion.

## Introduction

Rapid developments in smartphone and internet use have broadened people’s opportunities for the production and consumption of online content. Platforms and apps collectively termed social media, facilitate many-to-many communication in contrast to traditional one-to-one personal communication and one-to-many media communication [1]. Over the last decade, youth have been one of the biggest groups online [2]. In the United States, the proportion of adolescents who have a smartphone is estimated to be 89%, which has doubled over a 6-year period from 2012 to 2018; moreover, 70% of teenagers use social media multiple times per day, which is up from 30% in 2012 [3]. In Australia, a longitudinal study found that over 86% of students owned smartphones in grade 8, which increased to 93% by grade 11, with a marked increase in social media communication from teenage to early adulthood [4].

With this surge of online activity among youth, research into the relationship between social media use and health, especially among those in critical stages of development, such as the transition from teenage years to young adulthood, has gained importance [5]. Popular social media sites, such as Facebook, YouTube, Instagram, and Snapchat, provide extensive opportunities to youth to connect with others, express their creativity, and assert their self-identity through pictures, text, audio, or videos [6]. While the functionality and popularity of each platform varies, they are now well established as avenues for identity and self-presentation [7], building social capital [8], and participation in social activism [9] among youth.

Internet use and excessive social media engagement have, however, been linked to cyberbullying, social isolation, stress, and depression [10]. Frequent social media activity is also linked to harmful behaviors, such as addictions, self-harm, and suicidality that can be detrimental to mental health [11]. Although much of the narrative on social media implies an adverse effect on mental health, more recent research findings have been mixed. For instance, a review indicated that some uses of social media, such as suicide prevention [12] and authentic self-presentation, are associated with mental well-being in adolescents [5]. Mental well-being refers to several positive aspects of mental health, such as happiness, life satisfaction, positive relationships, positive outlook, and personal growth [13]. The positive impacts of social media are attributed to increased access to social capital and useful information that present various easy modes of social support in a virtual world [8]. These are of particular relevance among youth experiencing emotional problems who reportedly prefer anonymity offered by social media to in-person interactions [14].

Considering the centrality of social media in forming close connections, which contribute to positive youth development [15], and the high exposure to social media use among youth, understanding how social media influences positive mental health would be beneficial in improving youth mental health. However, the benefits of social media to youth mental health are not fully recognized. A scoping review of 79 studies investigating the association of social media use with mental health and well-being among adolescents found that three-quarters of the studies focused on psychopathology, with limited data on positive outcomes, such as well-being, happiness, and quality of life [11]. The authors also identified a gap in the understanding of “how youth themselves experience and perceive relationships between social media and mental health” and thus highlighted the value of qualitative research in gaining a deeper understanding into this relationship from youths’ perspectives. A recently proposed multidimensional model of social media use (MMSMU) explains the role of social media in relation to both beneficial and adverse effects on youth mental health but emphasizes the need for furthering research into its complex psychological implications [16]. In addition, given the disparate patterns of social media use and impacts among different age and racial groups, it is recommended to consider the unique psychological perspectives among target populations and sociocultural settings [6].

Singapore is a high-income nation in Southeast Asia with a population of 5.70 million, of which 4.03 million are citizens or permanent residents comprising Chinese (76.2%), Malays (15.1%), Indians (7.4%), and other ethnic groups (1.4%). Youth (age 15-24 years) constitute 11% of the local population. The 2016 National Youth Survey found that 42% of youth spent 10 hours or more daily on online activities [17]. The daily consumption of social media for networking, news, and entertainment also grew from an average of 65% in 2013 to 80% in 2016 in this population. The survey also found that the levels of life satisfaction and happiness have remained stable despite the increase in social media consumption. In Singapore, youth engagement in social media has been previously investigated in relation to psychological stress [18], suicidality [19], body esteem [20], daytime sleepiness [21], online bullying [22], and social activism [23]. However, to the best of our knowledge, the role of social media in positive mental health among youth is underexplored.

Given the lack of knowledge on the potentially beneficial links between social media and youth mental health in Singapore, this study explored lived experiences of youth on how social media contribute to positive mental health. Specially, we aimed to understand the components of and pathways to acquiring positive mental health from youths’ perspectives through thematic analysis and concept mapping.

## Methods

### Study Design

Following an interpretivist approach [24], we used qualitative semistructured interviews and focus group discussions (FGDs) to explore youths’ perspectives on the role of social media in positive mental health. This method enabled a deeper understanding into the subjective view and perceptions of youth.

### Ethics Approval

Ethical approval was obtained from the National Healthcare Group’s Domain-Specific Review Board (DSRB Reference 2020/00228). After discussing the aim of the study and the processes for safeguarding data and participants’ identities, all participants and parents of those under 21 years of age provided written informed consent.

### Sample

A purposive sample of youth, aged 15 to 24 years, was selected for the study. This age group was selected to reflect the age range for youth specified by the United Nations [25]. Including this age group was of particular interest to this study because these are critical transitional life stages (from teenage to adulthood and from being a student to being employed) and are the best periods to introduce mental health interventions. Table 1 contains information on the participants’ backgrounds. The sample was designed to include equivalent proportions of male and female participants, youth in the age ranges of 15 to 19 years and 20 to 24 years, and those belonging to the 3 main ethnic groups in Singapore (Chinese, Malay, and Indian), along with a smaller number of youth from other ethnic groups. This allowed collection of rich and balanced information from a diverse group of youth. Efforts were also taken to include participants with experiences of psychological distress, school drop-out, or risky behaviors, such as gang participation or substance use and incarceration, so that the findings represent a wider community of youth, who may not be in the academic setting, which seems to be the population in the majority of past research on youth mental health [11]. In order to recruit youth, the first few referrals were sought from colleagues and acquaintances, and the participants were provided with study brochures to disseminate to others and initiate snowball recruitment. Referrals were also sought from community-based youth welfare services.

**Table 1:** Participant background.

Variable			Focus group discussions (11 discussions; n=70)		Semistructured interviews (n=25)		Total (n=95)
Age (years), mean			20		21		20
**Gender, n (%)**							
	Female	37 (52.9)		14 (56.0)		51 (53.7)	
	Male	33 (47.1)		11 (44.0)		44 (46.3)	
**Ethnicity, n (%)**							
	Chinese	25 (35.7)		7 (28.0)		32 (33.7)	
	Indian	20 (28.6)		7 (28.0)		27 (28.4)	
	Malay	21 (30.0)		7 (28.0)		28 (29.5)	
	Others	4 (5.7)		4 (16.0)		8 (8.4)	
**Highest education level attained, n (%)**							
	Primary	1 (1.4)		2 (8.0)		3 (3.2)	
	Secondary	32 (45.7)		4 (16.0)		36 (37.9)	
	Junior college	13 (18.6)		10 (40.0)		23 (24.2)	
	Diploma	12 (17.1)		4 (16.0)		16 (16.8)	
	Institute of Technical Education	6 (8.6)		2 (8.0)		8 (8.4)	
	Tertiary (graduate/postgraduate degree)	6 (8.6)		3 (12.0)		9 (9.5)	
**Employment, n (%)**							
	Employed, full time	4 (5.7)		3 (12.0)		7 (7.4)	
	Employed, part time	6 (8.6)		6 (24.0)		12 (12.6)	
	Unemployed, never worked	18 (25.7)		4 (16.0)		22 (23.2)	
	Unemployed, past work/internship experience	42 (60.0)		12 (48.0)		54 (56.8)	

### Data Collection

Data were collected through online videoconferencing using the Zoom platform for all FGDs and 21 interviews. The other 4 interviews were conducted in person. Data used in this study belonged to a larger study exploring the meaning and pathways to positive mental health among youth. Participants were also specifically asked about their experiences with social media use and how social media had benefitted their mental health. For this study, “social media” broadly referred to any social networking website or app that enabled them to share and access information in the form of posts, photos, videos, messages, news, comments, etc [26]. Participants were encouraged to share experiences with any sites and platforms if they considered them to be relevant to positive mental health. This provided adequate flexibility in the content generated through the study. Key probes included in the interview/discussion guide are listed in Textbox 1. In addition, FGD participants were asked to provide multiple single-word responses to the question “When I use social media, I feel…” to act as quick starting points for the discussion. The interviews lasted for approximately 1.5 hours, while FGDs took 2 hours on average.

**Interview guide to understand the beneficial role of social media for youth mental health.**

**Interview guide**

- Thinking about places and practices youngsters are most exposed to nowadays, what are some of these that can influence their mental health?
- Which of these are beneficial to them/their mental health? And how?
- What about social media? How can social media benefit youth mental health?
- What about yourself? For example, in relation to social media how has this improved your mental health? Thinking about the past 1 year, can you give me some examples?
- What about your friends? Any pleasant or unpleasant experiences/incidents that you can recall in relation to social media? How does it make them feel?
- Activity (only for focus group discussions): “When I use social media, I feel…”
- When have you/your friends felt (experience/emotion)? Can you/someone describe any such incident or experience? How did it influence your/their mental health?
- Which aspects or components of positive/good mental health does social media influence? How?

These discussions were audio recorded with participants’ permission, transcribed verbatim, and anonymized. The content of the discussions was regularly reviewed by the research team to assess adequate data saturation and allow for identification of key themes. Data collection was discontinued after 11 FGDs and 25 interviews. Data from interviews and FGDs were combined to conduct pooled analysis.

### Data Analysis

Coding and data were managed with the NVivo 11 software [27]. In the initial stage, thematic analysis was undertaken through inductive coding, constructing categories, and continuously comparing codes between the transcripts and coders [28]. Preliminary codes representing broad categories on how social media benefitted mental health were created from the first 6 transcripts and discussed between coders, and based on their relevance to the research aims, refinements were incorporated. Codes were then grouped into wider categories to produce a coding framework [29], which was applied to the rest of the transcripts. The current analysis focused on positive influences of social media on youth mental health. The categories were reviewed, and key themes were generated from participants’ narratives on positive experiences with social media and their relation to positive mental health. Qualitative concept mapping [30] was conducted using the transcripts to identify short statements or phrases that were then linked in unrestricted “chain” sequences [31] to generate interpretable pathways to positive mental health in youth. This method has been previously used to conceptualize information relating to mental health from a public health perspective [32,33] and has been found to be useful “as an evidence-gathering tool” in research [34].

## Results

### Participant Background and Social Media Use

A total of 36 data units were included in this study, comprising 25 interviews and 11 FGDs. Participants’ details are presented in Table 1. There were 51 female participants and 44 male participants, and almost equal proportions of Chinese, Malay, and Indian participants, with a small number belonging to other ethnicities, such as Filipino and Burmese. Our participants included 8 youth with a history of psychological distress, school drop-out, or risky behaviors, such as gang participation, substance use, and incarceration.

All the participants had access to smartphones, and they used a wide array of social media platforms regardless of their demographic characteristics. These platforms included Instagram, TikTok, WhatsApp, Twitter, YouTube, Reddit, LinkedIn, Facebook, and others, with the first 3 being the most commonly quoted online platforms. They used social media platforms to create and follow online identities, communicate with friends and family, build social networks, and access information resources relating to news, fashion, hobbies, sports, health, and employment.

### Role of Social Media in Attaining Positive Mental Health

**Figure 1:**
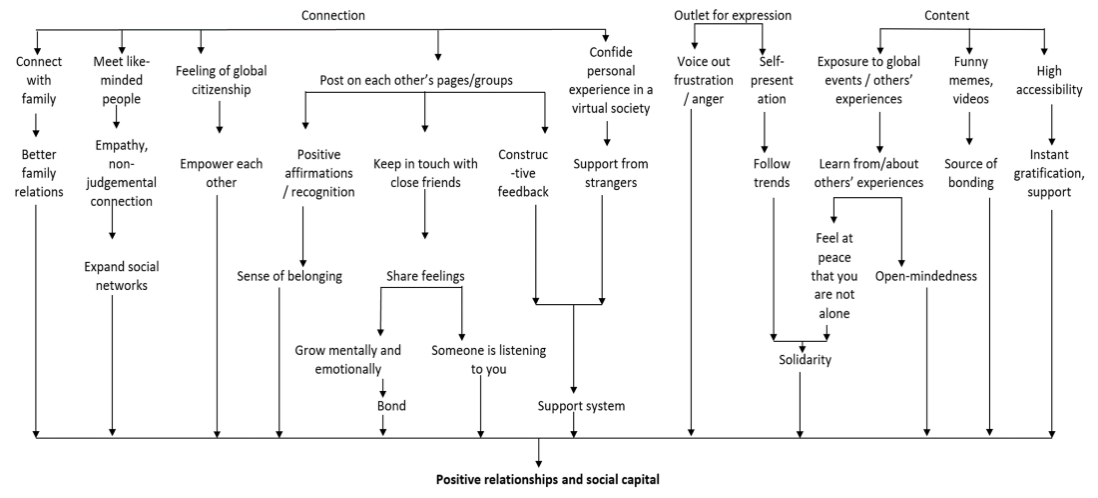
Social media as a way to build positive relationships and social capital.

**Figure 2:**
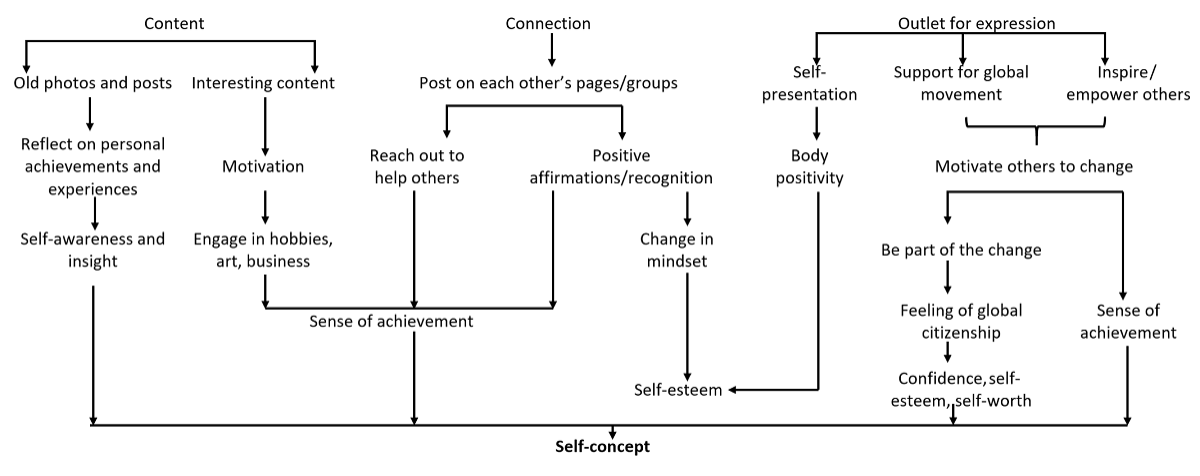
Enriching self-concept through social media.

**Figure 3:**
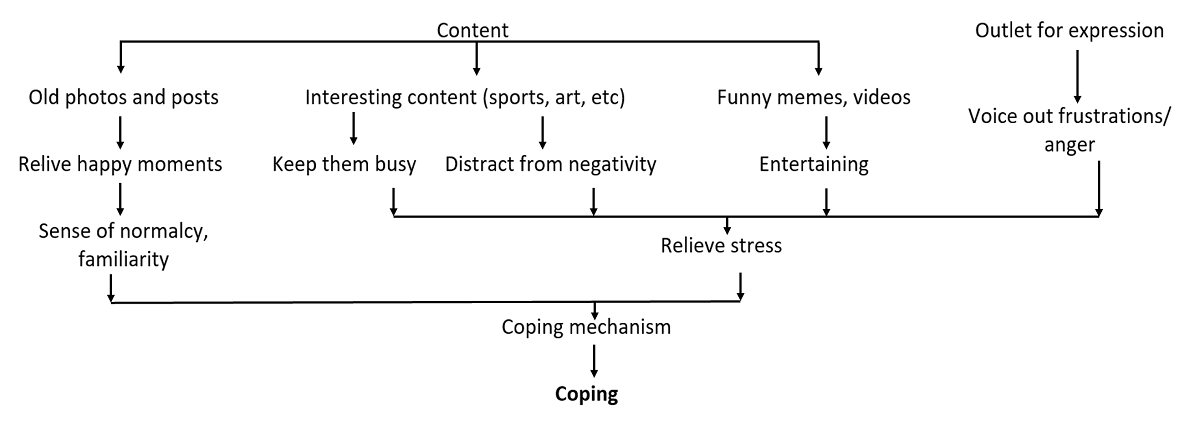
Social media improve the coping process.

**Figure 4:**
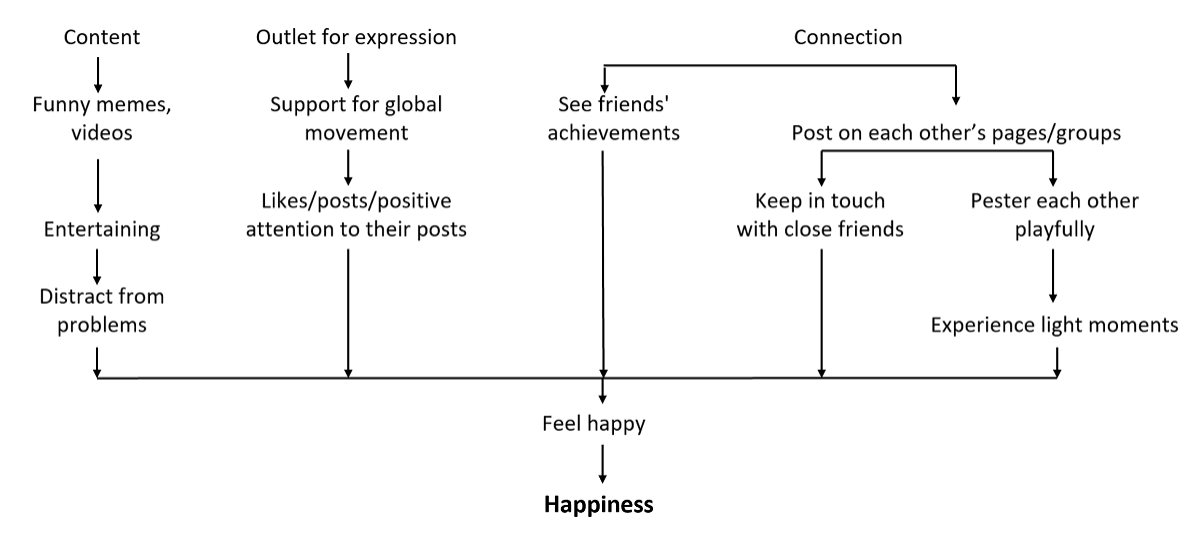
Feeling happy through social media.

**Figure 5:**
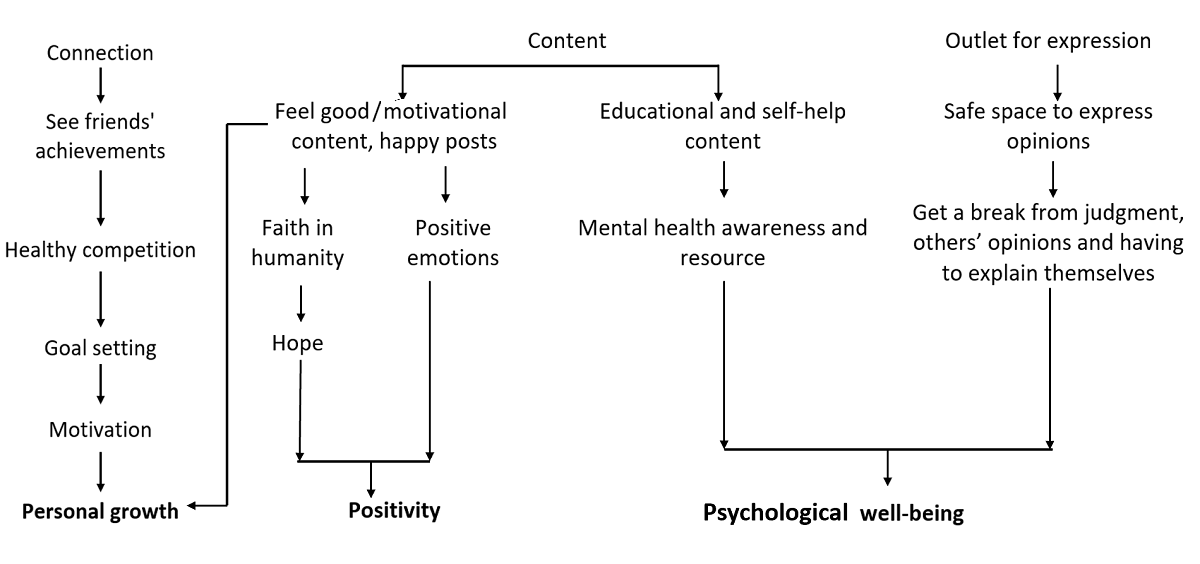
Influence of social media on other positive mental health aspects.

Based on participants’ narratives, our analysis suggested 3 features of social media consumption, namely, connection, content, and outlet for expression, with each influencing multiple aspects of positive mental health. These pathways contributed to the following 5 positive mental health components: (1) positive relationships and social capital (Figure 1), (2) self-concept (Figure 2), (3) coping (Figure 3), (4) happiness (Figure 4), and (5) other relevant aspects of mental health (positivity, personal growth, and psychological well-being) (Figure 5).

#### Theme 1: Social Media as a Way to Build Positive Relationships and Social Capital

Reflecting on the potential of social media as a mode to connect with others, participants discussed how they used different platforms to stay in touch with their family and communicate with friends virtually by posting messages on each other’s pages. These actions often resulted in developing close relationships with mutual trust, recognition, and a sense of belonging.

I guess when my friends tagged me in a photo and all that. Some of my friends, they're more appreciative and expressive on Instagram posts, so they will have this long message, especially after Uni camps or like Poly camps everyone will start like appreciation posts and all that. I guess that's the sense I feel included or appreciated, in that sense, though it's not extremely significant for me lah, but I guess in that sense being tagged in that message or on a photo on Instagram, on social media, it does make me feel included.

FGD participant #08

The participants often used social media as a channel to share feelings with their friends and felt that they always had someone out there who was listening to them and willing to provide constructive feedback and support.

I use Instagram to be very cathartic, so when I'm feeling very angry or stressed, Instagram has this feature where you can have a close friends’ group. So in that close friends group, I tend to rant and express my emotions, and then it's good because it's also another layer of a support system.

FGD participant #02

If I Tweet out, “I'm sad,” she can immediately send me ten memes rather than having to come directly over to my house or even like telephoning me by like being-- asking me directly, saying, “How are you feeling?” Like it's a-- sorry, no. I think it's also another way-- instead of calling me on the phone, which is also effective, it's an alternative also, another immediate alternative.

Semistructured interview participant #13

The youth also offered support and empathy to others who needed help. Through these, the participants narrated how they were able to grow close emotionally and bond with their peers.

I think apart from getting inspired or getting educated from other people, it gives ourselves a platform to do the same, as well. So for example… I also during the COVID [referring to Covid-19 pandemic] period, I also started out like a small business with my friend that... inspired [to donate] the profit… to donate it to other people. So that's kind of one of the positive change that we are trying to do, as well. So apart from getting educated from other people, we also are kind of inspired to do the same for others, as well.

FGD participant #02

For our participants, social media provided opportunities to expand their social network and meet like-minded people beyond their family and friends. Furthermore, they considered how they were able to confide their personal experiences in their virtual network and derive a feeling of support and global citizenship.

Social media is a platform like how you can find other people, like-minded people, you can see, if they're trying to connect with people who have the same beliefs, ideas, or struggles that you have, so you can connect with them and talk with them. You can share with them your beliefs without being heavily judged.

FGD participant #06

By expressing their feelings and preferences either anonymously or openly in the relatively nonjudgmental environment of social media platforms, our participants were able to bond with others who shared their values or sentiments, participated in popular trends, and showed solidarity with global movements, such as “Black Lives Matter” and climate change activism. They also often derived helpful knowledge from others’ experiences and felt that they were not alone when encountering difficulties.

I have seen opportunities across the spectrum where people empower one another and support good causes and stuff. So relating back to this question, I guess seeing the way people can empower and help one another, like the recent Black Lives Matter stuff, I've seen how people will help one another, linking donation links and stuff.

FGD participant #02

Besides the aspect of interaction that improved relationships between the youth and their local and global counterparts, our participants highlighted how social media were accessible 24/7 if they needed support or access to content such as memes, which they could share with their friends to bond with them and expand their social capital.

Memes….[where] people just put their words and stuff, and you need to have that context behind it. But it feels it's like a whole bonding thing for all the people in that age group. Everyone kind of has that similar experience or relatable stuffs. So it's kind of like everyone coming together. Yeah. They'll be, “I'm not alone in all of these things that we are going through.” It's a way for us to come together, despite the distance. It's not just people in a friend group, like 4 or 5 people. It's literally millions of teenagers around the world, who are all coming together like, “Yeah. Okay. Life sucks, but we're doing it together.”

FGD participant #05

#### Theme 2: Enriching Self-concept Through Social Media

Many of the participants narrated how they evaluated and perceived themselves while using social media platforms. It appeared to both reinforce and provide avenues for self-awareness and self-esteem. Some expressed how reviewing their older posts helped them gain insight into themselves, while others explained how presenting themselves in the way they truly were increased their self-acceptance, body positivity, and eventually self-esteem.

I think that's always very nice to be reconnected with someone and to kind of like start to share more about yourself. Like you say you've been up to and by doing that you kind of like also reflect on the achievements and things that have been happening in your life to see whether-- it kind of helps me kind of like have a little more insight on what I'm doing and how has that been helping me.

FGD participant #11

Self-esteem was also gained through their social connections who provided encouragement and recognition due to which some participants were able to appraise themselves more positively and gain confidence.

At one point, I felt pressured to create things that was basic or simple so that more people can buy it. But then I felt frustrated because that was not the reason why I started the business in the first place. So I posted about that on my Instagram, …[]...I was really actually speechless because I didn't even know these people and they showed me so much love. And it felt really good. Yeah. It helped me improve. Because I was feeling really down at that time, so it helped me become more confident and assured that I can do this.

FGD participant #07

Our participants described how social media helped them gain a sense of achievement through accessing positive content that motivated them to achieve more, be it pursing their interests or growing their careers. Some participants shared how they derived confidence and self-worth by being able to motivate their friends or lend support to others or events happening around the world.

I felt confident and good knowing that I do care about world issues in my own perspective because I care about world issues. So it made me felt good as a citizen. It made me felt good as someone as part of this world, that I don't not care about the world, but I care about the world. So it kind of made me feel better as a person.

FGD participant #10

#### Theme 3: Social Media Improve the Coping Process

From the narratives in our study, youth seemed to use social media to relieve stress in various ways. By being able to engage in relaxing activities or by expressing their frustrations or anger without being judged or shamed, our participants argued that it was a simple way to destress from problems.

I believe some of us are sacred to go to our parents and talk through some issues with our parents. So we choose instead to post on social media ... Because you get to rant it out on your [page]-- you get to rant it out either way. You get to express what you feel on a platform where you know you won't be judged. You know people won't shoot you down for it. At least you feel secure that-- you also feel secure that, one, they get to rant out their emotions. Two, they know that there are people who really care for them and want to know what's on their minds instead of just keeping it all in.

FGD participant #09

Participants also recognized that they benefited from the content available on social media that involved reliving happy memories or spending time entertaining themselves through watching light and humorous videos. These provided them with a sense of normalcy and familiarity in a changing world and helped relieve their stress. The youth often used these techniques to cope, rest, and recharge.

I started realizing that a lot of people started to look back at the memories. So for Instagram there’s this archive feature, ...where you can look back at your post like, years back. And whatnot. A lot of people started throwing back all these pictures so I don’t know where did that sense of familiarity or rather, that sense of normalcy, kind of helped them cope.

Semistructured interview participant #10

Young people also frequently accessed content related to their personal interests, such as sports and hobbies. The content helped them to engage actively and stay busy, distracting them from negativity and acting as a coping mechanism.

I think like itself as a platform for me to kind of distract myself because when I'm watching a video, I'm invested in what's going with video instead of whatever negativity that may be happening in life.

FGD participant #03

#### Theme 4: Feeling Happy Through Social Media

The participants identified social media as a source of happiness in their daily routines. While describing instances when participants felt happy, they indicated how it could be rooted in meaningful relationships they had built with their social networks. Having a positive self-image or experiencing positive affirmation through “likes” from friends or when they supported popular global causes promoted their happiness. Participants’ happiness was also enhanced when they cherished happy moments with their friends or participated in appreciating their friends’ successes.

I guess when my best friend posts her baking videos on her stories right, I always react very excitedly. I'd be like, “Oh my God. the buns look good.” Yeah, that kind of thing. So yeah. I don't know why but her baking and showing everyone the end product makes me very happy, not only just looking at food but really happy that she's finding something that she really enjoys doing. So yeah. That gets me excited.

FGD participant #01

Many of the participants used social media to distract themselves from stressors by consuming memes and funny videos on social media to feel happy. Our participants also often accessed motivational content to enhance their happiness.

TikTok videos for humor, I guess, it really helps you feel more happy. Recently discovered TikTok and there was 1 point of time, I think 2 months ago, I was pretty upset. So I downloaded TikTok, and I wasn’t expecting much. I was just thinking it’s some stupid app, stupid videos. But then I realized it was pretty funny and I was laughing for like 1 hour straight, and that made me feel happy. So I guess that’s the good thing about social media.

Semistructured interview participant #08

I think last year on Instagram, I started following a lot of these quotes pages and inspirational pages. And sometimes, every morning when I wake up, right, and before I see all the other celebrity posts or my friends going out and everything, sometimes I see these inspiring and really like motivational quotes. And I don't know. When I think about it and then I start my day off with something like that, then it just makes me happier, I guess. It just gives me that little boost to go about my day.

FGD participant #06

#### Theme 5: Influence of Social Media on Other Positive Mental Health Aspects

Given that youth spend a considerable amount of time using social media, they described how the materials they access are beneficial to their overall mental health and well-being. Our participants viewed social media as a safe space that facilitated open expression of their emotions and experiences. This allowed them to take a break from others’ judgement and avoid feeling the need to constantly explain themselves, unlike what might be expected of them in environments under adults’ command.

I think the aspect of open-mindedness is much more influenced by social media. Because I think since social media is very-- it's very large. And it basically is exposed to-- I mean, almost all youth, right? And I think social media in itself is-- it's basically giving people the voice to speak up. So it's very unrelenting in that way because it accepts everyone's opinions, everyone's views, on topics and stuff like that. And it just exposes everyone to that. And I think being exposed to various topics and viewpoints and opinions, it sort of creates that open-mindedness quality in youth where they think where now it's more the norm to just accept other people's opinions I would say. Because it just become more normal to listen to other people's opinions and be nonjudgmental about it I would say.

Semistructured interview participant #15

Few participants also shared how seeing their friends’ growth presented them with an opportunity for healthy competition and motivated them to raise their personal goals. This was deemed important as setting goals for themselves was recognized as an important aspect of positive mental health among youth. Others also shared how they grew their skills through coaching and educational content accessed on their social media platforms.

The competitive side, yes, is good, …there is positive competition to outdo yourself, like outdo your friends.

FGD participant #06

People learn a lot of tips and tricks online, something like, how to cook, how to make this, how to solve this, how to do financial stuff in Singapore (be)cause we are not taught to do that in school. So we learn it online and we educate ourselves online.

FGD participant #06

Another benefit of using social media was access to feel-good and motivational content that could promote mental well-being by introducing participants to resources, which some participants mentioned improved awareness on their personal mental health. Specifically, through access to happy posts and motivational content, some participants were able to experience more positive emotions, hope, and positivity.

I think that's one thing about social media like, okay, you can also spread negativity, but at the same time, you can use that platform to spread positivity also. ...- so I have these friends who also post small happiness, like small bundles of joy that they experience and it could be like some aunty smiling at them when they are crossing the road or all those kind of small things. And sometimes, when you see those, then your faith in humanity is just restored. You're like, “Okay, maybe life is quite good.” And there are lots to look forward to. So in that way, that's also another way of how social media is so hard to give up. Because sometimes a lot of people also focus on the negativity of social media. There's also so much of positivity that comes with it. It all depends on how you view it.

FGD participant #06

## Discussion

Increasing levels of internet and social media penetration in the daily lives of youth have resulted in significant influences of social media practices and culture on mental health among youth. Our study provides new insights into the ways youth can achieve and experience positive mental health through social media. Our analysis suggested 3 features (content, connectivity, and modes of expression) offered by social media as beneficial to positive mental health among youth. Five main themes were identified, namely, positive relationships and social capital, self-concept, coping, happiness, and other relevant aspects of mental health, such as positivity and personal growth, offering an in-depth understanding into the different pathways through which social media can affect positive mental health.

These routes are partly consistent with the MMSMU of Yang et al that proposed pathways to youth well-being resulting from their activities, motivators, and communication on social media [16]. The MMSMU was theoretically developed from past cross-sectional quantitative research. Thus, our qualitative study sheds further light on the important links between social media use and positive mental health using the lived experiences of youth contemporary to a generation of heightened technology users. Specifically, our study showed how attributes of social media, such as the content available on them, social connections formed on social media, and outlet for self-expression, lead to positive mental health through multiple interconnected pathways.

The relevance of positive mental health to positive relationships, social support, and social networks, often referred to as social capital, has been well established. According to the model of psychological well-being by Ryff [35], positive relationships with others relate to the establishment of close, trusting, and meaningful bonds with others, and being able to reciprocate by showing empathy and support to others. Research has shown that good relationships with friends and family in adolescence buffer the stresses of teenage life [36] and serve as early sources of emotional support and coping that can have long lasting impacts on the life course [37]. Our results indicate that social media can improve relationships and enhance youths’ social capital, likely through mutually satisfying and close interactions with peers and by expressing their support on every day and global matters. The concept of solidarity, in combination with the aspects of social cohesion and capital, has gained prominence with the increase in social media–related activism [23]. Social activism can form integration, identity, and ties in a community, be it physical or virtual, and bind people to one another. It can also influence psychosocial processes by providing youth with a source of meaningful connection and mutual respect, thus increasing their sense of belonging and purpose in life [38]. Our results lend further support to this notion and indicate that social media can serve as a tool to enable youth to connect with a social community, thereby benefiting psychologically from the gained social capital.

Based on the experiences of our participants, we uncovered several mechanisms that develop and strengthen self-concept among youth. Self-concept refers to how people perceive themselves physically, socially, academically, or professionally. Youth is a critical age for the development of positive self-concept, which has shown long-term benefits to individuals [39]. Constructing profiles and receiving social feedback on social networking sites were associated with adolescents’ self-esteem and achievement of self-concept clarity, which in turn were related to positive self-appraisal and a strong sense of personal identity [40]. Clarity of self-concept is also associated with better self-knowledge, personal goals, and relationships [41]. It is also considered vital to psychological well-being, particularly of young people, as people who feel good about themselves and their abilities are known to be more happy, motivated, and successful and have lower risks of depression and anxiety [42]. Social media sites empower users to take an active role in constructing their own self-identity [43]. Authentic self-disclosures can produce greater intimacy among peers [44] and lead to social support and autonomy [16]. Research has also shown that self-disclosure helps by getting feedback from peers and develops a sense of self [45]. Our study supported these results strongly and found that youth partake in self-disclosures through posts to show solidarity with global causes and seek support from their friends. Through social media, they also aim to receive attention from a broad spectrum of people in their virtual community in direct or indirect ways, and in doing so, enhance their self-worth and self-esteem. In addition, our study identified the value placed on older posts that can help youth reflect on their personal achievements and gain self-awareness, which has not been previously reported in research.

Coping involves a collection of behavioral and cognitive responses aimed at minimizing the effect of stressors [46]. Recent research on the physiology of stress mechanisms has shown the potential of social media in reducing biological stress responses, such as heart rate and cortisol production, with exposure to social media immediately after acute stress induction compared to other activities such as reading [47]. Studies also indicate that social support obtained from online relationships can be beneficial in coping. For example, youth are likely to discuss their mental health problems on social media [48] or connect with strangers who may be enduring similar problems [49]. Similarly, a recent study found that support from virtual communities is as effective as face-to-face support in terms of coping with stress [50]. There is also evidence that when adolescents received “likes” and positive affirmation for their posts, it resulted in reward processing that could help modulate brain areas involved in stress responses [51]. Our findings developed through qualitative enquiry are consistent with the findings from these previous studies and provide a single framework on how social media could benefit individuals seeking support and avenues for relaxation from stressors. The results from this study thus add to the literature on social media influence on coping mechanisms among youth and provide potential anchors for assessing and reducing the stress they experience.

In this study, our participants narrated several instances when they felt happy while using social media or used social media to feel happier. Happiness or subjective well-being is defined as a positive evaluation of how one’s life is progressing [52]. It is associated with higher life satisfaction, quality of life, and self-esteem, and better academic outcomes in adolescents and young adults [52,53]. In relation to social media and the culture of self-presentation on popular platforms among youth, such as Instagram [3], it is believed that happiness is dependent on the cognitive and affective processes involved in drawing social comparisons with peers [54]. Comparisons on social media are more often associated with a negative body image and poor life satisfaction than positive mental health [5]. The relationship between social media and happiness can thus vary. However, a recent study found that the impact of social comparison through social media on happiness depends on the type of platform or the activities engaged by youth [54]. For example, platforms that relied heavily on visual appeal, such as Facebook, were found to reduce happiness, while writing or reading blogs was found to have a positive effect on life satisfaction and overall happiness. Contrary to the current literature on the adverse impact of comparing with peers [54], our study participants mentioned that they felt happy to see their friends’ achievements. It is possible that they were only referring to close friends, and it made them appraise their own life positively. In fact, much of the experiences relating to happiness were linked to close friends or community bonding, sharing light moments, and entertainment associated with social media, and it is possible that in an Asian setting, positive relationships influence the experience of happiness on social media. Future quantitative research should investigate our framework statistically to further understand the process of happiness in relation to social media.

Our participants also alluded to other components of positive mental health where social media featured prominently. These were related to deriving a sense of positivity, avenues for personal development and growth, and other ways in which social media were generally beneficial for participants’ mental health and well-being. Creating environments with positive experiences and increasing competence have been indicated as relevant to positive youth development [15]. In relation to social media, positivity can result from news feeds and posts that make individuals feel good and inspire them to achieve more. Strategies to get into the habit of being motivated to embrace positive change and being able to contribute to others’ motivation have been widely adopted lately by youth to tackle the negativity associated with social media [53]. Research findings also indicate that prioritizing positivity as opposed to the pursuit of happiness is associated with more positive emotions, self-compassion, resilience, and less depressive symptoms [55]. Given the limited research in this area, our findings provide a foundation for understanding the role of social media in youth positivity and mental well-being.

The strengths of this study include the generation of rich qualitative data with a large purposively selected sample of youth and the use of an online activity within the FGDs to prompt reflection on social media experiences and facilitate a rich discussion. This is also the first study to have exclusively investigated positive mental health among youth in a community sample comprising a population of teenagers and young adults, thus reflecting their experiences at important developmental milestones. However, the study also has some limitations. Our research does not provide a detailed account of the ways in which different social media platforms are experienced by youth in relation to their mental health or whether the frequency of use and patterns of use have any bearing on their experiences. Our study also lacks a deeper exploration of the negative context and experiences with social media that are often associated with depression and anxiety in youth [56]. In addition, participants’ reflections on specific social media sites and online practices were grounded in their day-to-day experiences, which may be subject to rapid development in preferences and platforms. It is therefore necessary to review the findings in the context of technological developments in the future. Finally, our study sample mainly consisted of Asian ethnic groups living in a high-income country with high internet and smartphone access. The findings may therefore not be generalizable to youth residing in low-income and non-Asian settings.

In conclusion, our study results highlight the integral role of social media in the lives of today’s youth and indicate that social media can offer opportunities for positive influence, personal expression, and social support, thus contributing to positive mental health among youth. Among Singapore’s youth, improved positive relationships and social capital, self-concept, coping, happiness, and other aspects, such as positivity and personal growth, are linked to self-expression, connections, and content on social media platforms that youth are exposed to. Our study indicates that social media have been used by youth for increasing connectivity, broadening social networks, and increasing knowledge, and for entertainment purposes. Advancements in digital and health technology have also prompted interest in social media as a potentially inexpensive way to implement mental health promotion, impart psychoeducation, and reduce stigma [57]. Our study findings could inform public health policies or mental health promotion measures focusing on interventions in youth who are connected to social media use in schools or other settings. Specifically, interventions can promote the ways in which social media engagement could bring about positive mental health, such as improving self-esteem and supportive social networks among adolescents and young adults. Furthermore, information on social media can be tailored to youth based on their priorities and value systems [58]. Our findings can thus be applied to inform and optimize engagement with youth through social media, and enhance digital modes for mental health promotion and intervention.

## Abbreviations

FGD: focus group discussion
MMSMU: multidimensional model of social media use
